# Short-term relief or long-term solution? A comparison between endoscopic stenting and gastrojejunostomy for malignant gastric outlet obstruction

**DOI:** 10.1186/s13017-026-00709-8

**Published:** 2026-06-10

**Authors:** Stephanie Cheng, Xin Yan Lim, Crystal Jin Yang Sia, Gek Hsiang Lim, Jeremy Tian Hui Tan, Beatrice Fangju Koh

**Affiliations:** 1 Department of General Surgery, Singapore General Hospital, Singapore, 169608 Singapore; 2 Health Services Research Unit, Singapore General Hospital, Singapore, 169608 Singapore; 3 Department of Upper Gastrointestinal and Bariatric Surgery, Singapore General Hospital, Singapore, 169608 Singapore

**Keywords:** Gastric outlet obstruction, Gastrojejunostomy, Endoscopic stenting

## Abstract

**Introduction:**

Malignant gastric outlet obstruction is the consequence of advanced cancer resulting in mechanical obstruction to gastric emptying. Traditionally, surgical bypass (GJ) is performed and is known for its durability but also for its high morbidity. In contrast, endoscopic stenting (ES) is less invasive option but carries a notable risk of stent dysfunction and the need for subsequent reinterventions. These patients usually have limited life expectancy with reduced quality of life. The aim of this study is to review the different treatment options and compare their safety and efficacy.

**Method:**

We performed a systematic review and meta-analysis comparing GJ to ES for malignant gastric outlet obstruction (mGOO). Comprehensive search of electronic databases between January 2001 and December 2022 was performed to identify relevant studies. The primary outcomes assessed were length of stay, reintervention rate, procedure-related complications and secondary outcome was mortality.

**Results:**

32 articles were abstracted in this meta-analysis with a total of 3296 patients. The re-intervention pooled risk ratio was 0.34 (95% CI 0.22, 0.52), indicating the risk of reintervention was lower among patients who underwent GJ. However, the results were comparable between ES and GJ for procedure-related complications and mortality. The length of stay was higher among those who underwent GJ, with a weighted mean difference of 11.2 days (95% CI 4.4, 18.1).

**Conclusion:**

GJ bypass was associated with lower risk of re-intervention and comparable mortality and procedure-related complications compared to ES in patients with malignant gastric outlet obstruction, despite also being associated with longer length of stay.

## Introduction

Malignant gastric outlet obstruction (mGOO) is a common complication of advanced malignancies of the upper gastrointestinal tract, usually due to intrinsic or extrinsic blockage of the stomach or duodenum. Distal gastric, rarely duodenal cancers can result in intraluminal obstruction in late stages and rare malignancies like ampullary, cholangiocarcinoma can result in this condition as well. Patients with mGOO often present with nausea, vomiting and occasionally can also experience abdominal pain, poor oral intake causing poor nutrition, dehydration and weight loss. These symptoms significantly impact patients’ quality of life and hence palliative therapy for mGOO is crucial [1].

The aim of palliation is to allow re-establishment of oral intake and minimise post-procedural complications at the same time. Surgical gastrojejunostomy bypass has been traditionally [2] used to manage mGOO. However, surgical bypass has been associated with complications like delayed gastric emptying, prolonged post-operative hospitalization stay and post-operative mortality [3]. With most of these patients presenting with advanced disease, a majority of them have limited life expectancy and some of them might not be suitable surgical candidates, mostly due to frailty associated with poor oral intake and hence significant weight loss and poor physiological reserve. Endoscopic placement of self-expanding metal stents (SEMs) provides these patients with another alternative to surgery [3].

The past decade has seen the introduction and widespread use of enteral stents [3–5] to manage these patients. Several studies showed that ES has a high success rate [6–8] and can allow faster relief of symptoms as compared to GJ. ES is also associated with a shorter hospitalization stay [4, 9] and lower procedural related mortality rate [10–12] hence making it a promising and less invasive alternative for patients with mGOO.

Our study aims to review the present literature comparing ES and GJ in the management of malignant GOO in terms of re-intervention rate as our primary outcome and also to evaluate procedure related complications, length of hospital stay and post-procedure mortality rates.

## Method

Two independent reviewers conducted systematic search in the PubMed and Embase databases to identify relevant studies. The search terms will include Medical Subject Headings (MeSH) terms and keywords related to “gastric outlet obstruction,” “endoscopic stenting,” “gastrojejunostomy,” and “malignancy.” Boolean operators (AND, OR) will be used to combine search terms appropriately.

The titles and abstracts of the identified articles are assessed for eligibility for inclusion based on the predefined criteria. Full-text articles of potentially eligible studies will be retrieved for further assessment.

Inclusion criteria included articles in English, studies involving human subjects, published between 1 January 2001 to December 31, 2022 and studies comparing endoscopic stenting and surgical gastrojejunostomy for the treatment of malignant gastric outlet obstruction. Study types included randomized controlled trials, cohort studies and case-control studies. Articles in languages other than English, non-human subjects or insufficient data were excluded. Systematic reviews and meta-analyses and conference abstracts without full-text articles were also excluded.

The risk of bias in studies were assessed with ROBINS-I (Risk of Bias in Non-Randomized Studies—of Interventions) tool for cohort studies, and RoB 2 tool for randomized controlled trials (RCTs). Quality assessment was conducted independently by two reviewers.

Relevant statistics (mean, standard deviation, proportion and sample sizes) pertaining to the reintervention rate, length of stay, mortality rate and procedure-related complication rates were extracted from articles documenting the effect of bypass and stent on patients diagnosed with pancreatic cancer. Heterogeneity in the studies was first examined based on Higgin’s I2 statistic and Cochran’s Q-test, and where heterogeneity was assessed to be significant, pooled estimates would be reported using a random effects model based on DerSimonian and Laird method. Otherwise, pooled estimates would be reported based on a fixed effects model using inverse variance method. Forest plots were generated using the available information extracted from the studies. Publication bias was assessed by using inverted funnel plot symmetry. Subgroup analyses were performed for the subgroups of laparoscopic and open GJ.

All analyses were performed using STATA 17 meta package. Statistical significance was declared if a 2-sided* p*-value < 0.05.

The overall quality of evidence was evaluated using the GRADE (Grading of Recommendations Assessment, Development, and Evaluation) approach. The results of this meta-analysis were reported in accordance with the Preferred Reporting Items for Systematic Reviews and Meta-Analyses (PRISMA) guidelines.

## Results

**Fig. 1:**
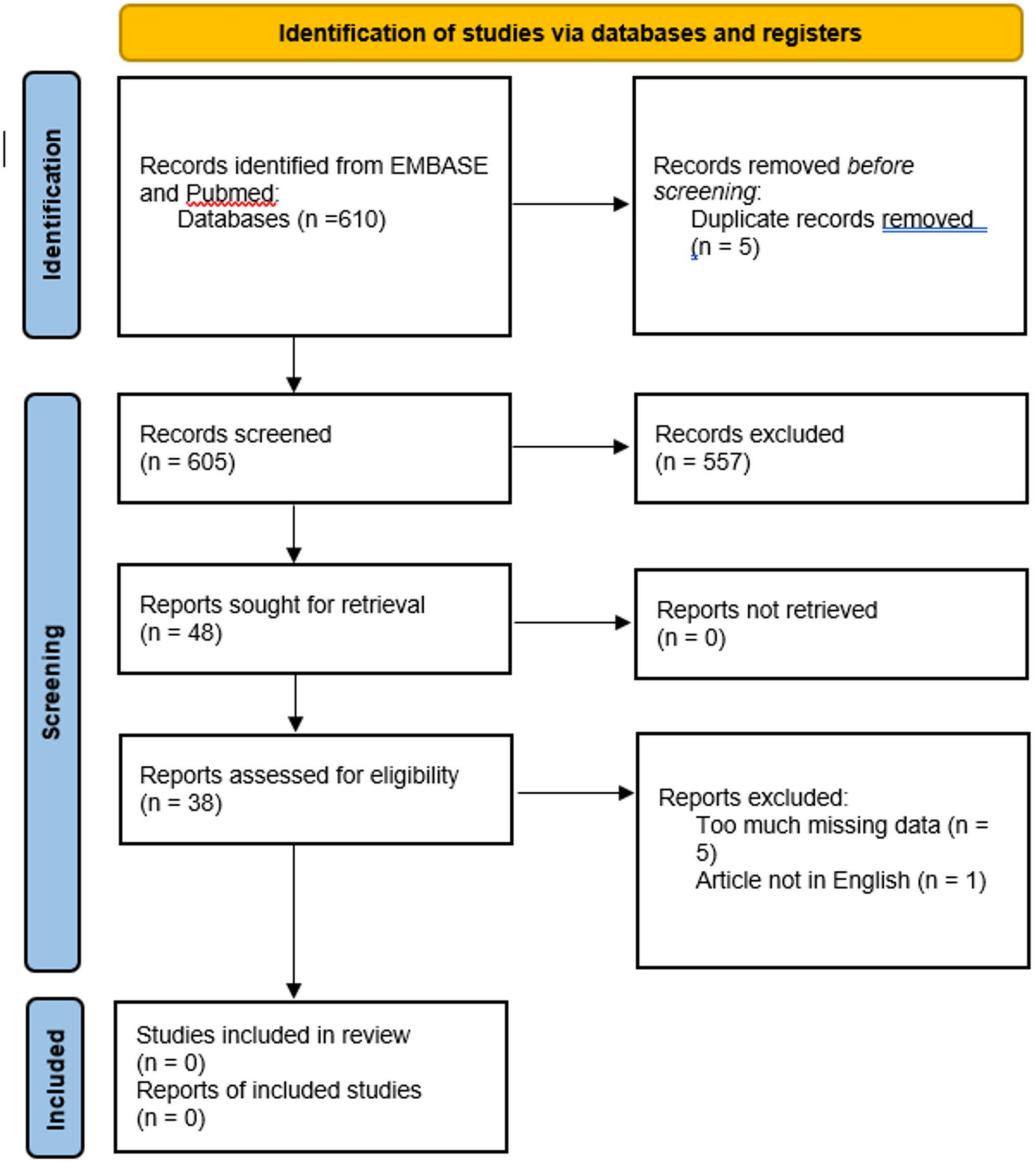
Identification of eligible studies for the meta-analysis comparing the outcomes of gastrojejunostomy and endoscopic stenting for gastric outlet obstruction

We identified 32 eligible studies to be included for full-text review in this meta-analysis. Total of 605 titles were screened after initial search from Pubmed and Embase databases and 48 were identified. Subsequently 32 were selected for full-text review. The selection process is showed in Fig. 1 with the characteristics of the 32 eligible studies summarised in Table 1.

**Table 1:** Characteristics of studies included in meta-analysis comparing SEMS and GJ bypass

Author	Year of publication	Country	Study period	Study type	Patients (*n*)		Age		Surgical approach			Types of malignancy		
					SEMS	GJ bypass	SEMS	GJ bypass	Open	Lap	Open and Lap	Gastric	Pancreatic	Others
Ovalle et al.	2022	South Africa	2010–2019	Cohort study	40	28	61	66	28	–	–	42	0	6 Biliary 20 others
Laitamaki et al.	2022	Finland	2015–2020	Cohort study	42	46	72	66	–	–	46	11	41	36 (12 Biliary, 4 colorectal, 9 duodenal and 11 not specified)
Jang et al. 2019	2019	USA	2011–2017	Cohort study	183	127	66.8	66.2	–	–	127	85	149	76 (metastatic)
Yoshida et al.	2017	Japan	2010–2016	Cohort study	23	30	70	63.5	–	–	30	–	53	–
Tamura et al.	2022	Japan	2016–2020	Cohort study	73	54	72	66	Not specified			–	127	–
Uemera et al.	2018	Japan	2008–2017	Cohort study	64	35	72	68	35	–	–	-	99	–
No et al.	2013	South Korea	2001–2010	Cohort study	72	41	66.4	64.	–	–	41	113	–	–
Park et al.	2015	South Korea	2005–2012	Cohort study	217	39	60.7	61.7	–	–	39	526		
Fisher et al.	2019	USA	2012–2015	Cohort study	134	193	62	65	–	–	193	72	185	70 (duodenal)
Roy et al.	2012	USA	2007–2008	Cohort study	29	75	59.6	62	75	–	–	Hepatobiliary, small bowel – numbers not specified		
Fiori et al.	2004	Italy	2001–2002	RCT	9	9	72	70	Not specified			Not specified		
Singh et al.	2013	USA	2000–2011	Cohort study	241	93	63.3	61.2	Not specified			Not specified		
Jeurnink et al.	2010	Netherlands	2006–2008	RCT	21	18	66	66	Not specified			3	28	8 (4 Duodenum, 1 biliary, 1 ampullary, 2 metastatic)
Lopez-Sanchez et al.	2019	Spain	2007–2018	Cohort study	28	30	78.1	69	30	–	–	35	10	13 (7 Duodenal, 3 ampullary, 2 metastatic, 1 retroperitoneal)
Piano et al.	2005	Italy	1997–2002	Cohort study	24	23	74	76	23	–	–	7	33	7 (1 Biliary, 4 metastatic, 2 papillary)
Wong et al.	2002	USA	1988–1998	Cohort study	6	17	Not specified	Not specified	17	–	–	23	-	-
Jang et al.	2017	South Korea	2009–2013	Cohort study	99	45	58.8	58.9	45	–	–	144	-	-
Khashab et al.	2013	USA	2001–2010	Cohort study	120	227	64	66	Not specified			Not specified		
Maetani et al.	2005	Japan	1994–2004	Cohort study	22	22	72.3	66.	22	–	–	44	–	–
Mehta et al.	2006	United Kingdom	Not specified	RCT	13	14	70.4	67.6	–	14	–	4	15	8 (2 Cholangiocarcinoma, 1 gallbladder, 4 metastatic, 1 benign gastric)
Johnsson et al.	2004	Sweden	1994–2004	Cohort study	21	15	80	69	15	–	–	19	7	10 (3 Biliary, 3 duodenal, 4 peritoneal)
Mittal et al.	2004	New Zealand	1989–2002	Cohort study	16	30	63.5	OGJ: 67.5 LGJ: 68	56	14	–	10	13	23 (1 Cholangiocarcinoma, 6 duodenal, 1 renal, 6 colonic, 1 lymphoma, 4 benign, 3 ampullary, 1 squamous)
Yim et al.	2001	USA, Singapore	1996–1999	Cohort study	12	15	Not specified	Not specified	Not specified			Gastric, duodenal, pancreatic, metastatic – numbers not specified		
Espinel et al.	2006	Spain	1999–2004	Cohort study	24	17	79	75.29	17	–	–	9	23	9 (2 Biliary, 3 duodenal, 2 ampullary, 2 gallbladder)
Maosheng et al.	2001	Japan	1983–1999	Cohort study	19	41	67.5	66	Not specified			–	60	–
Chandrasegaram et al.	2012	Australia	1998–2008	Cohort study	26	19	67	65	19	–	–	7	20	18 (12 Duodenal, 6 metastatic)
Haga et al.	2020	Japan	2015	Cohort study	85	94	71	71	47	47	–	179	–	–
Keranen et al.	2013	Finland	1999–2010	Cohort study	50	21	73	69	21	–	–	71	–	–
Shuai et al.	2018	China	2008–2014	Cohort study	29	34	64.6	59.8	–	34	–	54	–	9 (6 Bowel, 3 metastatic)
Yukimoto et al.	2018	Japan	2010–2016	Cohort study	38	27	73	75	Not specified			40	–	25 (Hepatobiliary)
Maetani et al.	2004	Japan	1993–2002	Cohort study	20	19	71.8	68.7	19	–	–	–	26	13 (11 Gallbladder, 1 biliary, 1 ampullary)
El-Shabrawi et al.	2006	Austria	2001–2004	Cohort study	22	17	75	76	17	–	–	11	16	12 (3 Duodenal, 5 biliary, 2 breast, 1 colonic, 1 undifferentiated)

**Fig. 2:**
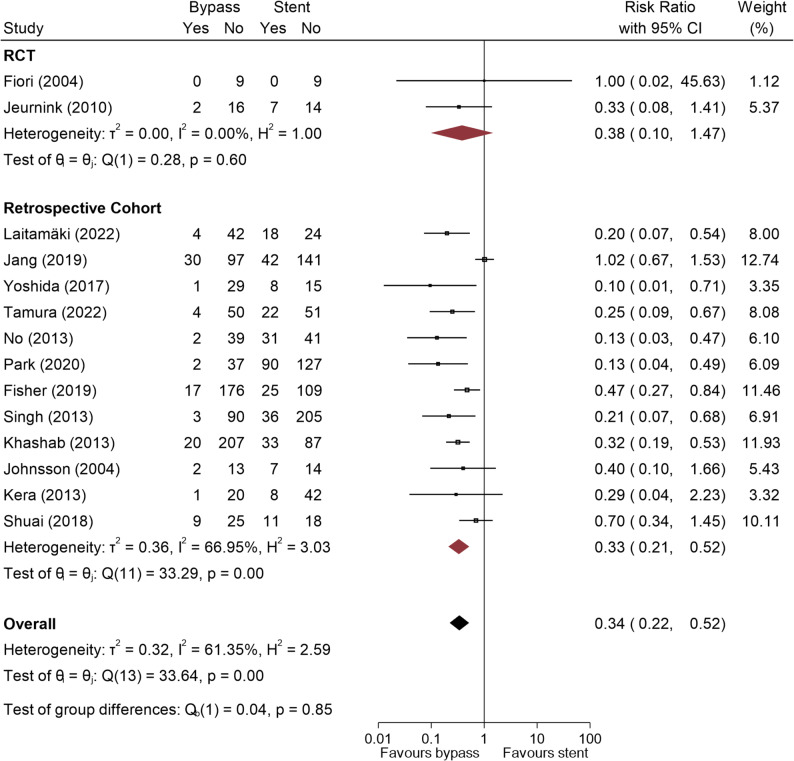
Forest plot comparing re-intervention between bypass and stent

The outcomes that were measured from the studies included were re-intervention rate, mortality, procedure-related complications and length of stay (LOS). Fourteen studies were included in this analysis of re-intervention risk (Fig. 2). A random effects model was used in view of the statistical heterogeneity observed in the studies. The pooled risk ratio was 0.34 (95% CI 0.22, 0.52), indicating that the risk of reintervention was lower among patients with bypass.

**Fig. 3:**
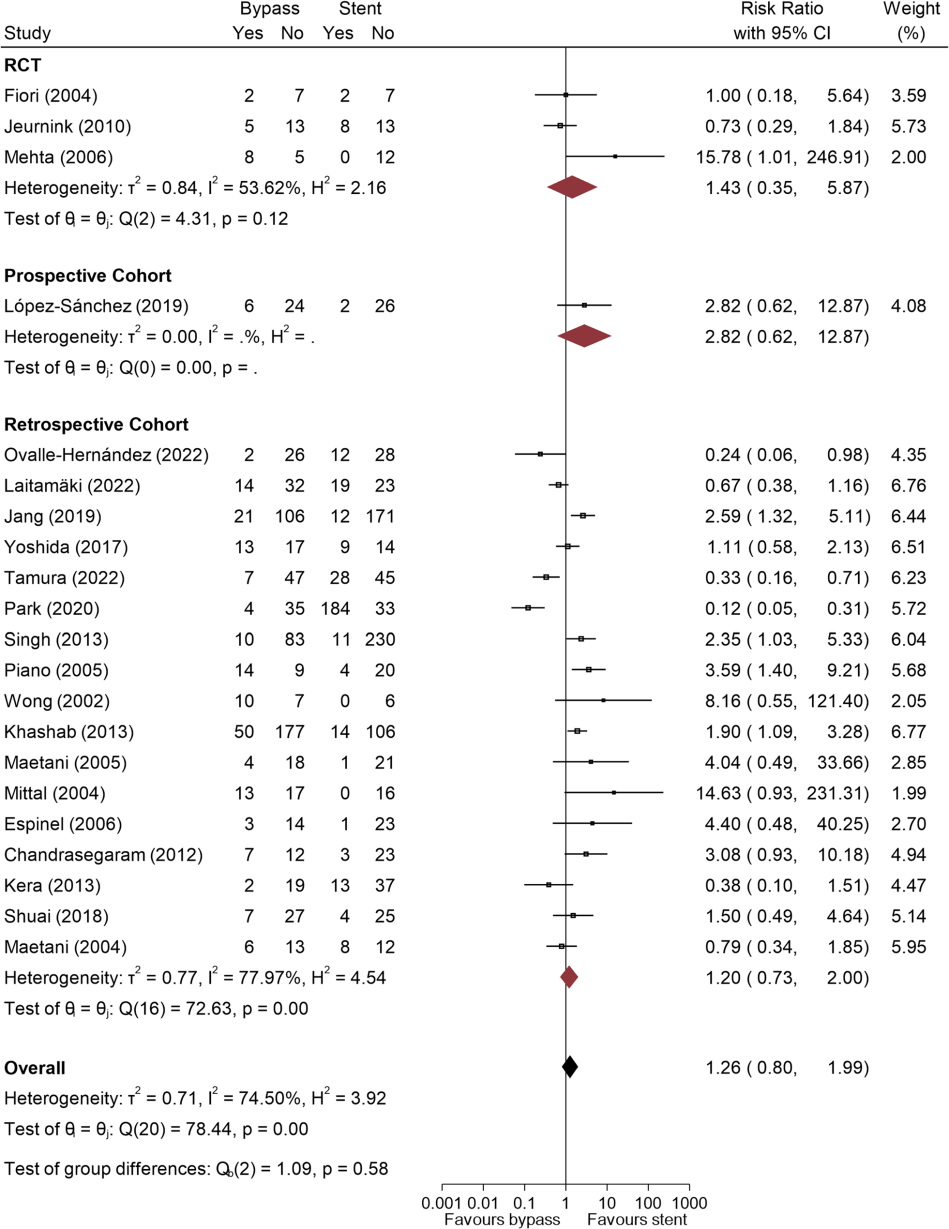
Forest plot comparing procedure-related complications between bypass and stent

21 studies were included in the analysis comparing procedure-related complications between GJ and ES (Fig. 3). A random effects model was used in view of the statistical heterogeneity observed in the studies. The pooled risk ratio was 1.26 (95% CI 0.80, 1.99), suggesting no significant difference between procedural complications between the two groups.

**Fig. 4:**
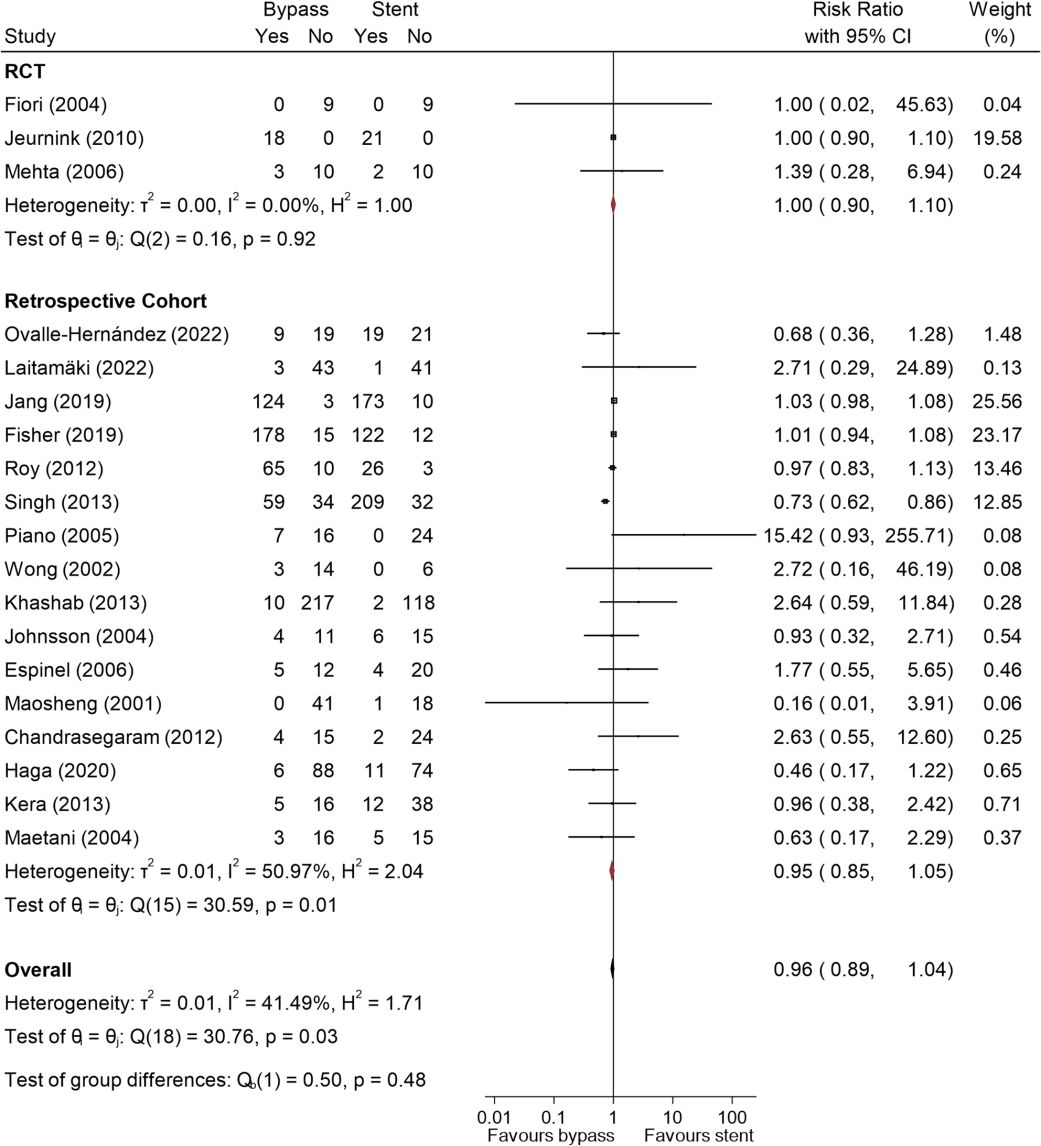
Forest plot comparing mortality between bypass and stent

19 studies were included in the analysis of mortality between patients who underwent ES and bypass (Fig. 4). A random effects model was used in view of the statistical heterogeneity observed in the studies. The pooled risk ratio was 0.96 (95% CI 0.89, 1.04), indicating that there was no significant difference in terms of mortality.

**Fig. 5:**
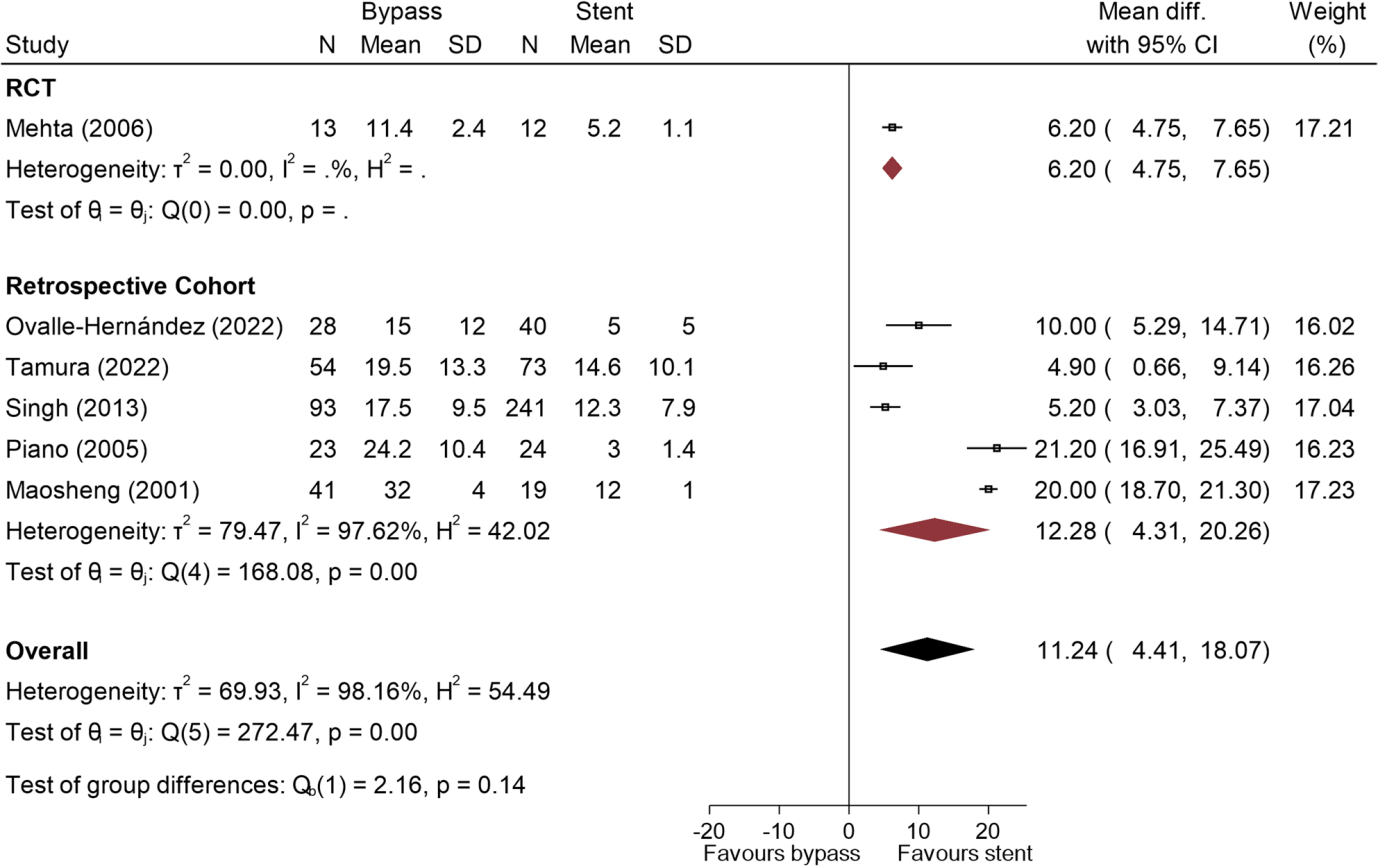
Forest plot comparing LOS between bypass and stent

Six studies were studied to compare the LOS between bypass and stenting (Fig. 5). In view of the heterogeneity observed in the studies (I^2^ = 98%), a random effects model was used. The weighted mean difference was 11.2 (95% CI 4.4, 18.1), indicating that the LOS was higher among patients with bypass.

**Fig. 6:**
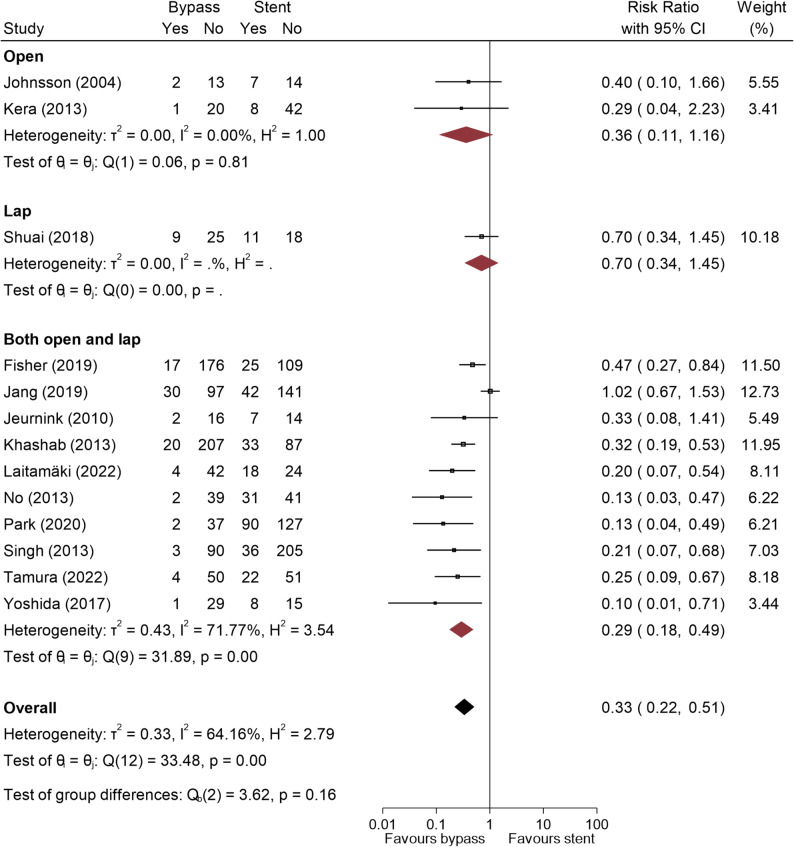
Meta-analyses of re-intervention rate, subgroup by approach of GJ

**Fig. 7:**
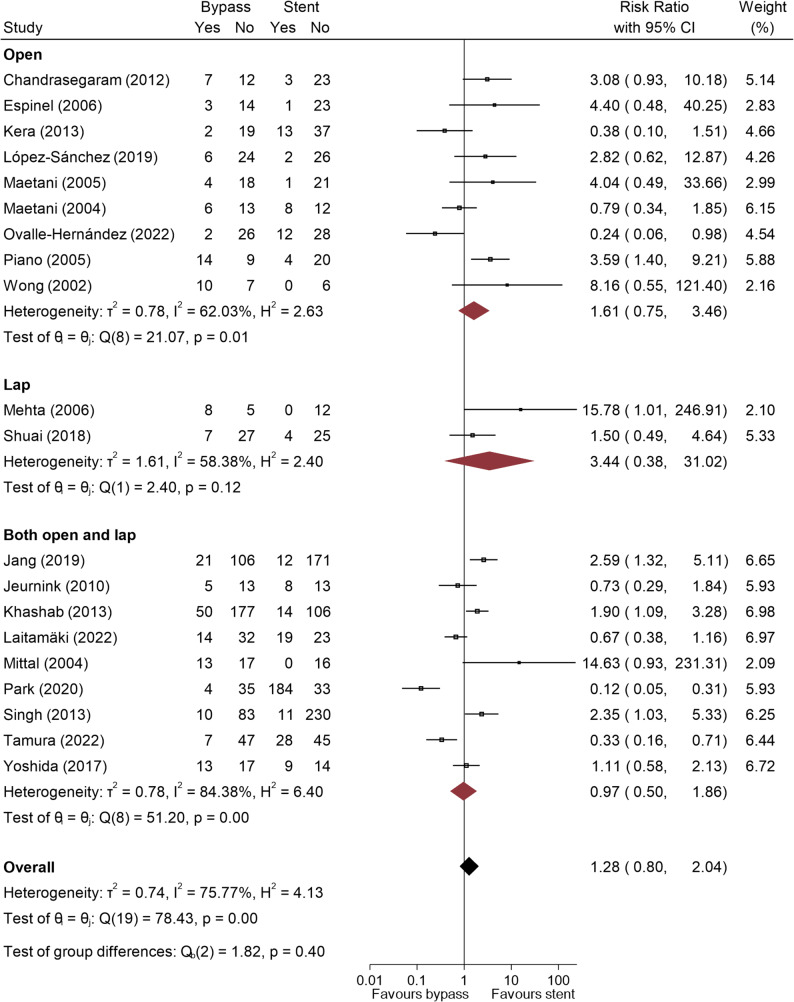
Meta-analyses of procedure-related complications, subgroup by approach of GJ

**Fig. 8:**
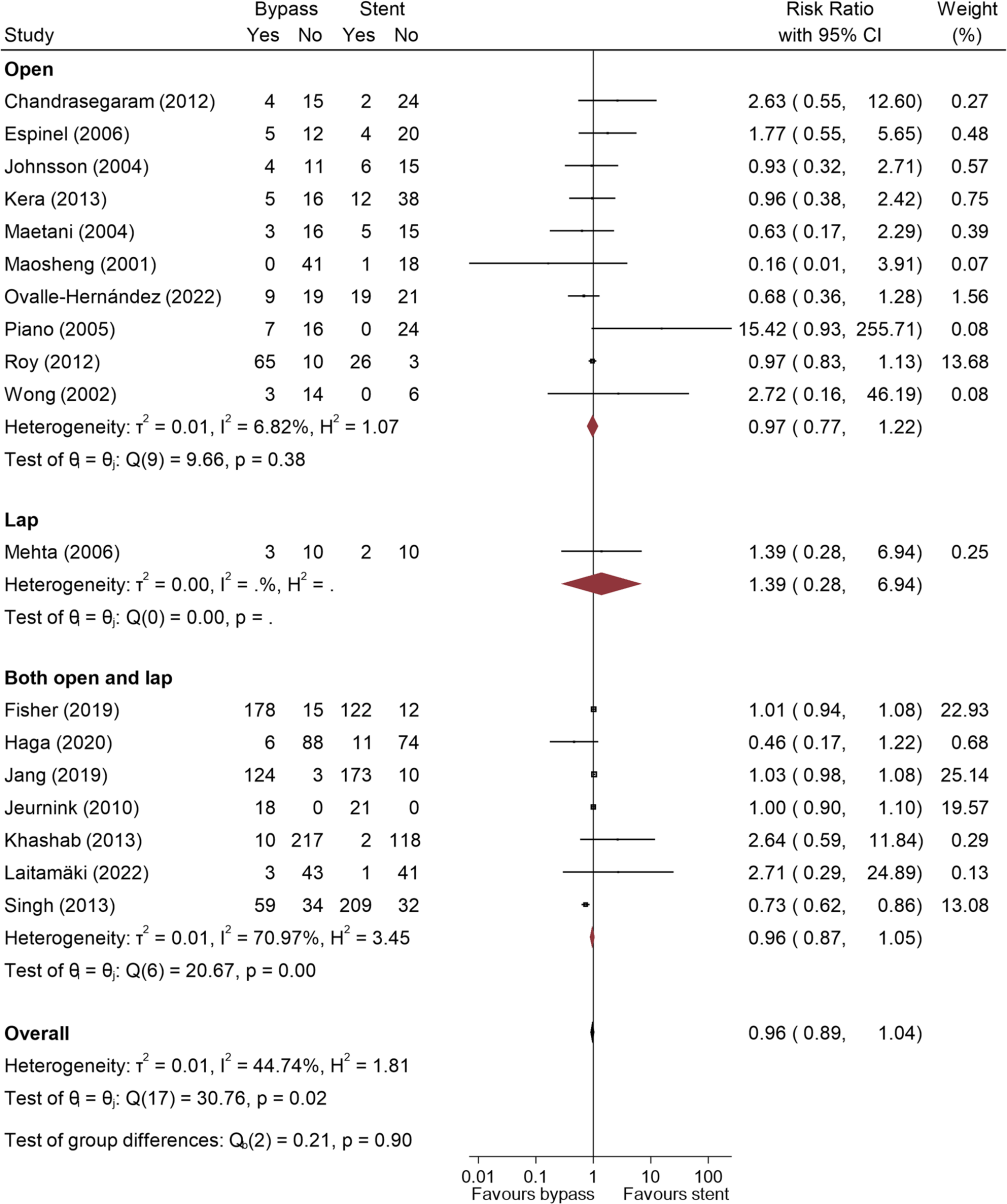
Meta-analyses of mortality, subgroup by approach of GJ

**Fig. 9:**
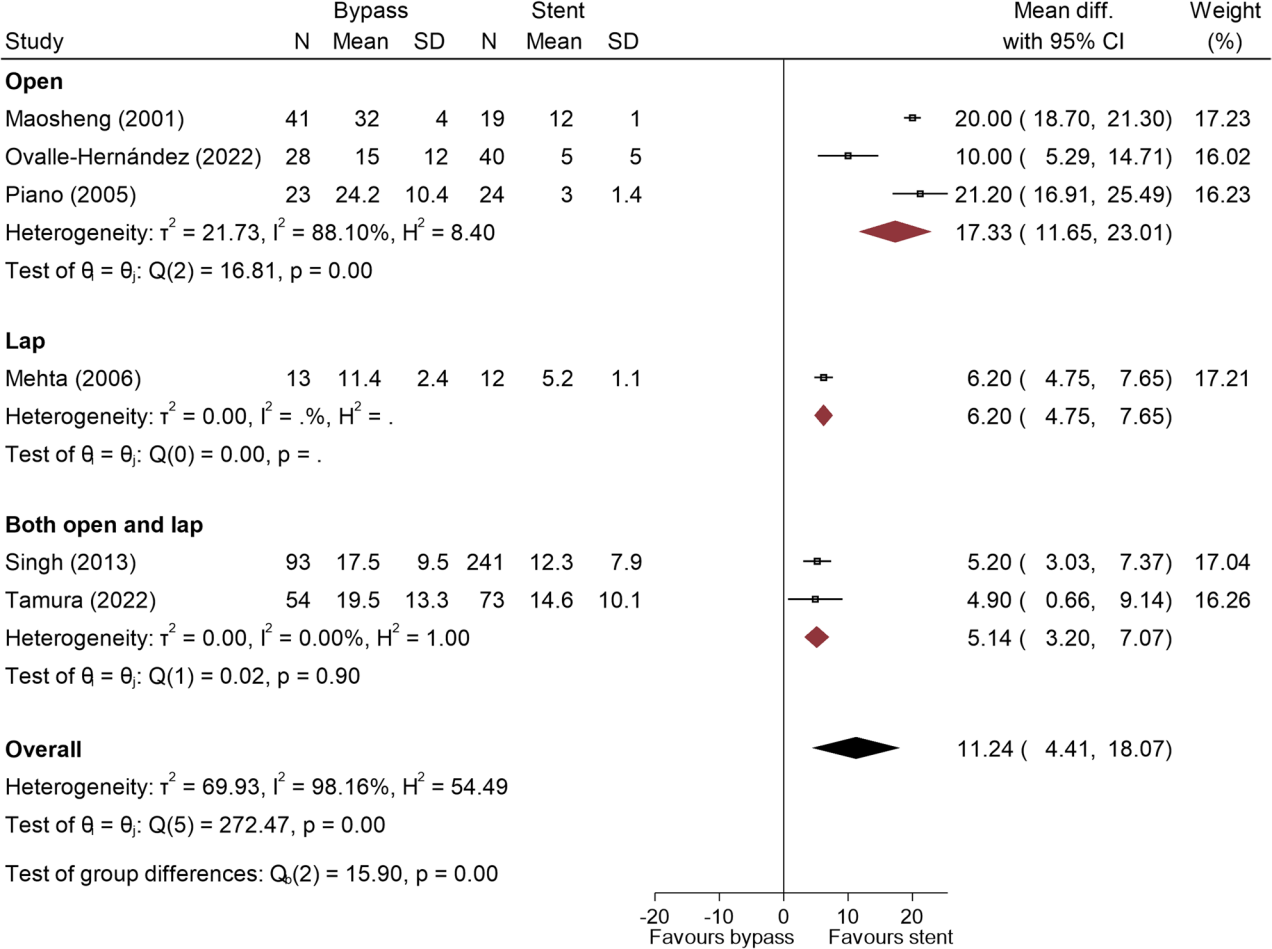
Meta-analyses of length of stay, subgroup by approach of GJ

In order to address heterogeneity found in our study, subgroup analyses were performed for laparoscopic versus open approach of GJ (Figs. 6, 7, 8 and 9). There is no significant difference in outcomes for re-intervention rate, procedural complications and mortality in the subgroup analyses, which may be due to a small sample size in each group, limited number of studies and heterogeneity in study populations.

**Fig. 10:**
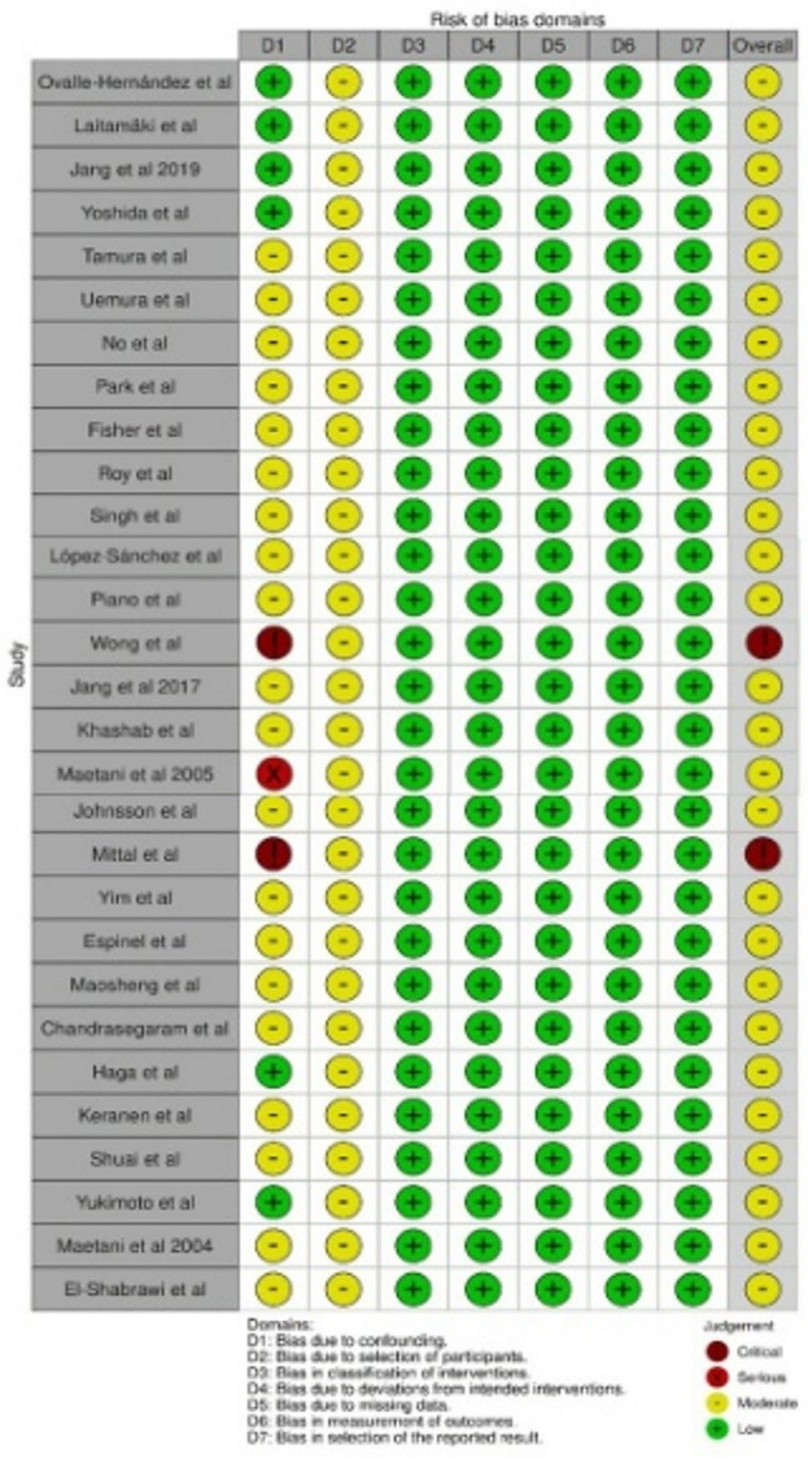
Risk of bias diagram for cohort studies

**Fig. 11:**
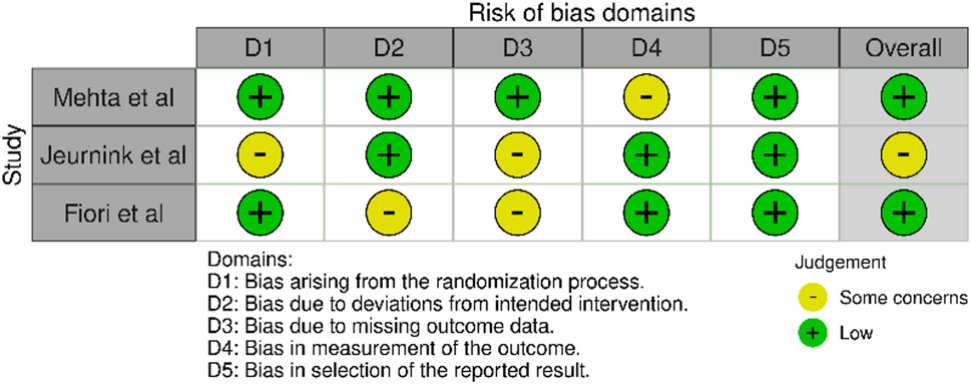
Risk of bias diagram for RCTs

**Fig. 12:**
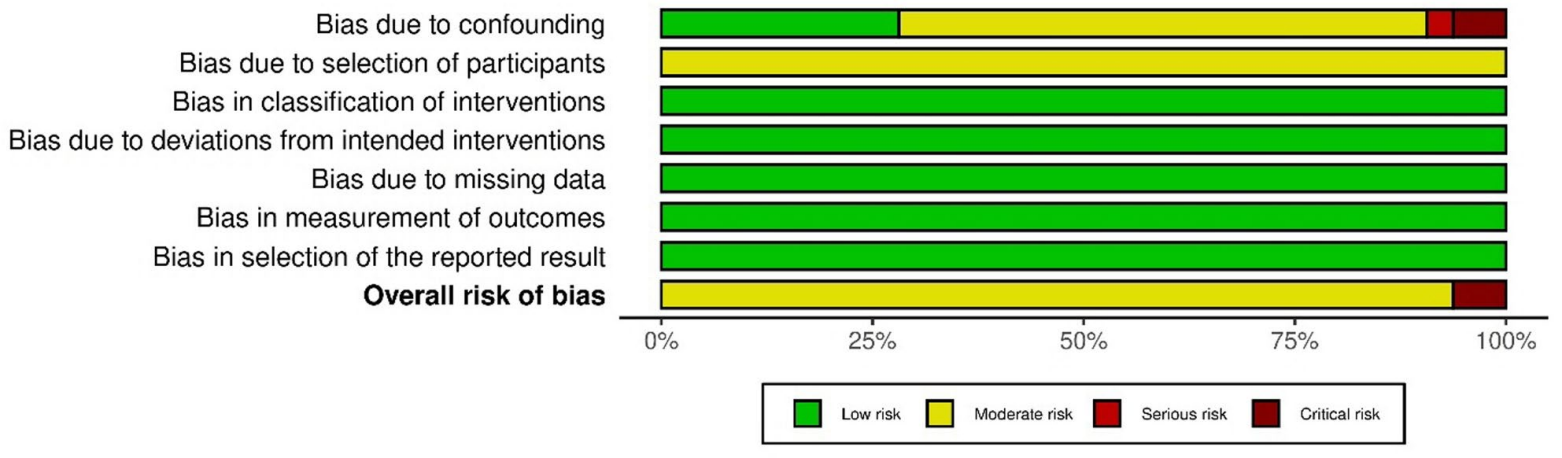
Summary of overall risk of bias for cohort studies

**Fig. 13:**
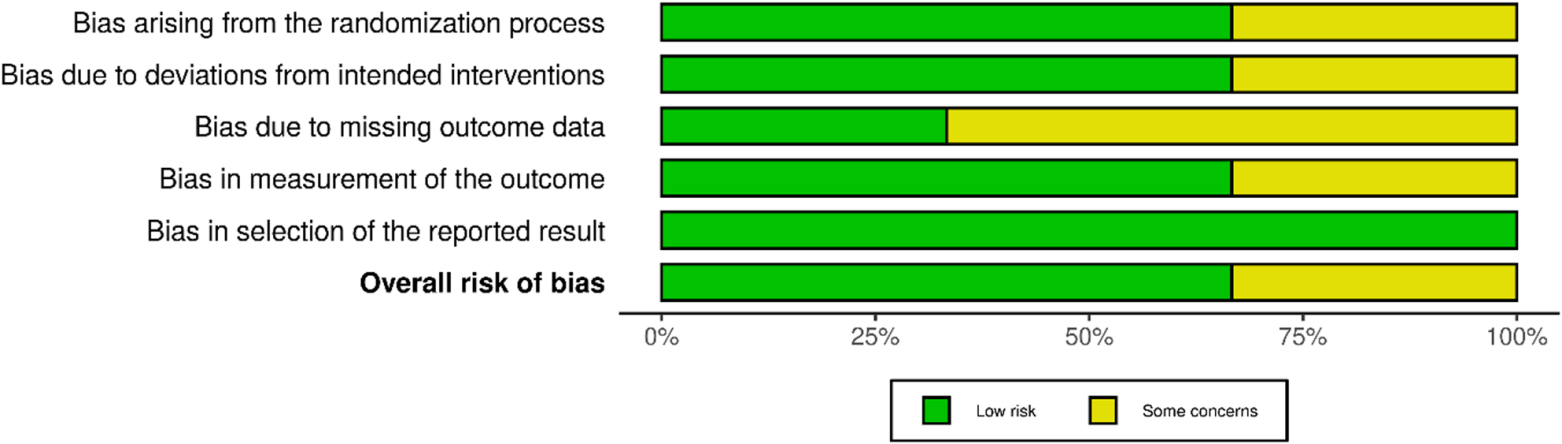
Summary of overall risk of bias for RCTs

The risk of bias was assessed by ROBIN-I and RoB 2 tools and summarized in Figs. 10, 11, 12 and 13. [13].

## Discussion

The management of malignant gastric outlet obstruction (GOO) is a critical aspect of care for patients with advanced upper gastrointestinal malignancies. In this systematic review and meta-analysis, we aimed to provide a comprehensive evaluation of the comparative efficacy and safety of endoscopic stenting versus surgical gastrojejunostomy as palliative interventions for malignant GOO.

Among the malignant causes of GOO, pancreatic cancers predominate [14]. It generally implies poor prognosis with median survival between 3 and 6 months, and the intent of treatment focuses on restoration of oral intake and relief of symptoms like nausea, vomiting, electrolyte imbalances and dehydration.

While malignant gastric outlet obstruction (GOO) significantly impairs patient quality of life, recent comparative evidence on the safety and efficacy of the two treatment modalities remains limited. Our study aimed to evaluate the effectiveness of gastrojejunostomy and endoscopic stenting by comparing re-intervention rates as primary outcome. Length of stay, complication rates and procedure-related mortality were assessed as secondary outcomes to determine the safety profiles of these interventions.

Both GJ bypass and endoscopic stenting have been previously proven to be safe and effective in terms of technical success; there is ample literature comparing GJ bypass and endoscopic stenting supporting this [3, 12, 15]. However, existing evidence often focused on overall survival (OS) and technical success, for example, Hong et al. [16] in their meta-analysis included 31 articles before 2021 compared technical success as well as overall survival, which showed both GJ and ES to have good technical success but OS favoured GJ. The study attributed differences in OS to be due to higher re-obstruction and re-intervention rates in the ES group, which in turn may have caused a shorter patency duration and hence poorer nutritional status and increased risk of developing GOO- related co-morbidities. In another meta-analysis by Miller et al. [17], they compared endoscopic ultrasound-guided gastroenterostomy (EUS-GE) to GJ and ES for mGOO and their results showed that EUS-GE had higher clinical success with significantly fewer adverse events compared to the other treatment options. However, it is important to note that the studies included had small sample sizes and both benign and malignant GOO cases were included in patient selection.

Prior meta-analyses had also often used clinical success as their primary outcome, and it was often defined variably e.g. time to oral intake, symptom scores or resolution of obstruction [16–18]. This heterogeneity introduces outcome-measurement bias and limits interpretability of any pooled effect on clinical success, even when the overall odds ratio appears null. Not only that, clinical success is also often measured early and captures only short-term relief, when outcomes between GJ and ES could lead very different long-term courses. Therefore, we believe re-intervention rate is a more objective, patient-centred and durable endpoint. The need for additional procedures, be it endoscopic re-stenting, surgical revision or percutaneous drainage, indicate failure of initial palliation with ongoing symptom burden and worse quality of life. The use of re-intervention rate is hence less time-sensitive, less heterogenous and thus comparable across studies and techniques.

### Re-intervention rate and length of stay

Our analysis indicates that GJ required fewer subsequent re-interventions, but was associated with a longer hospital length of stay. On the other hand, endoscopic stenting is associated with a shorter hospital length of stay but a higher re-intervention rate [3, 4, 16, 19–21]. In the SUSTENT study [4], patients who undergone ES had earlier tolerance of oral intake (5 vs. 8 days in GJ group) but issues such as stent migration or occlusion often required them to return for repeat procedures, potentially impacting the overall cost-effectiveness and patient satisfaction associated with stent placement. While GJ offers a lower risk of reintervention, its longer hospital stay and need for general anaesthesia may limit its suitability for frail patients. In contrast, endoscopic stenting, though associated with a higher reintervention rate, is less invasive, allows quicker recovery, and may better align with palliative goals of minimizing hospital burden and optimizing quality of life.

### Complications and Adverse Events

Our analysis revealed that there is no significant difference in mortality or complication rate between surgical GJ and stenting. In existing literature, GJ was associated with more major complications. Major complications are defined as severe, life-threatening events, including, but not limited to, haemorrhage, perforation, and peritonitis. In contrast, minor complications refer to non-life-threatening issues, such as mild post-procedural discomfort and vomiting. In our analysis, we did not differentiate between major or minor complications as there is heterogeneity in the classification of the severity of complications. These findings show both GJ and endoscopic stenting are safe approaches in the management of malignant GOO.

Overall, patient selection is paramount when considering between GJ bypass and endoscopic stenting. With a lower re-intervention rate for GJ bypass suggesting greater durability, this suggests it is more suitable for patients with better prognosis. Multiple studies have shown that the mean survival following enteral stent placement is approximately 12 weeks [3, 18, 22]. Hence patients with poorer prognosis or performance status should be offered SEMS placement especially since it is associated with a shorter hospital stay and earlier return of ability to tolerate orally.

### Study limitations

This meta-analysis has several limitations. Firstly, fewer studies were included in comparing LOS between GJ bypass vs. SEMS placement as majority of the papers did not provide mean and standard deviation in their LOS data. As such, these studies were excluded as it was not possible to evaluate variability. Besides, most of the studies either focused on open GJ bypass or did not differentiate between laparoscopic vs. open approaches, which could introduce further bias as open surgery usually necessitates longer hospital stay due to various reasons such as increased postoperative pain, increased risk of complications such as wound infections and ileus as well as slower recovery.

Secondly, the included studies exhibited heterogeneity in terms of study design, patient characteristics, and outcomes assessed. We have performed subgroup analyses in order to address this. Additionally, the potential for publication bias cannot be entirely ruled out, as unpublished or negative studies may not have been included.

Another limitation of this study is the exclusion of newer techniques such as EUS-guided gastrojejunostomy. Although promising, this approach remains technically challenging with significant variability in technique and a lack of procedural standardization, making meaningful comparison with well-established options like endoscopic stenting or surgical bypass difficult at this stage.

Given the escalating burden of healthcare expenditure globally, cost considerations should be integrated into clinical decision-making when evaluating treatment options for mGOO. Endoscopic stenting has been shown in several studies to offer lower initial costs and hospital stays compared to GJ but the higher re-intervention rates may have an impact on patients’ overall health status [4, 7, 23]. The SUSTENT trial [4] attempted a cost-utility analysis, despite its small sample size, showing that GJ had a marginally higher incremental cost-effectiveness ratio (ICER) when compared to ES. A recent study by Ramai et al. [24] endoscopic stenting cost USD$22,748 and yielded 0.31 QALYs, while EUS-GE cost USD$32,254 with 0.53 QALYs gained. Despite the higher cost, EUS-GE was deemed cost-effective with an ICER of USD$41,994 per QALY, falling below standard willingness-to-pay thresholds.

## Conclusion

In conclusion, our systematic review and meta-analysis provide valuable insights into the comparative safety of endoscopic stenting and surgical gastrojejunostomy for the management of malignant gastric outlet obstruction. Both interventions offer effective symptomatic relief and clinical success as shown in existing evidence, but they are associated with different re-intervention rates and length of stay profiles. Treatment decisions should be guided by individual patient characteristics to determine the most appropriate intervention. Future studies should aim to identify patient subgroups that may benefit more from each approach, assess long-term outcomes including survival, and incorporate local utility values, cost-effectiveness analyses, and patient-reported outcomes to support more holistic and patient-centred management.

The decision regarding whether to pursue endoscopic stenting or surgical gastrojejunostomy should be made within the context of shared decision-making, taking into account the individual patient’s prognosis, preferences, and potential risks and benefits associated with each procedure. Additionally, ongoing advances in endoscopic gastrojejunostomy techniques and stent design may influence the landscape of interventions for malignant GOO in the future.

## Data Availability

Not applicable.

## Abbreviations

ES: Endoscopic stenting
GJ: Gastrojejunostomy
GOO: Gastric outlet obstruction
LOS: Length of stay
mGOO: Malignant gastric outlet obstruction
OS: Overall survival
SEMs: Self–expanding metal stents
